# Novel c.2216T > C (p.I739T) Mutation in Exon 13 and c.1481T > A (p.L494X) Mutation in Exon 8 of *MUT* Gene in a Female with Methylmalonic Acidemia

**DOI:** 10.1007/s12013-013-9532-9

**Published:** 2013-03-12

**Authors:** George Imataka, Osamu Sakamoto, Hideo Yamanouchi, Shigemi Yoshihara, Yuki Omura-Hasegawa, Go Tajima, Osamu Arisaka

**Affiliations:** 1 Department of Pediatrics, School of Medicine, Dokkyo Medical University, 880 Kitakobayashi, Mibu, Shimotsuga, Tochigi, 321-0293 Japan; 2 Department of Pediatrics, Tohoku University School of Medicine, Sendai, Miyagi Japan; 3 Department of Pediatrics, Saitama Medical University, Saitama, Japan; 4 Department of Pediatrics, Shimane University Faculty of Medicine, Izumo, Shimane Japan; 5 Department of Pediatrics, Graduate School of Biomedical & Health Sciences, Hiroshima University, Hiroshima, Japan

**Keywords:** Methylmalonic acidemia, l-Methylmalonyl-CoA mutase, Vitamin B12

## Abstract

We report herein a 1.5-year-old girl with methylmalonic acidemia (MMA) in whom two missense mutations were found: a novel I739T mutation located in exon 13 and the L494X mutation in exon 8. The results of organic acid test showed a pronounced increase in methylmalonate excretion with increased methylcitrate and 3-OH-propionate excretion, leading to a diagnosis of MMA, and Vitamin B12 administration was started. Analysis of the *mut* gene confirmed a T-to-A substitution at nucleotide position 1481 in exon 8 and a T-to-C substitution at nucleotide position 2216 in exon 13, leading to the amino acid isoleucine at position 739 being changed to threonine, resulting in c.2216T > C (p.I739T). The patient has now been on high-dose oral administration of Vitamin B12 and carnitine therapy (900 mg of levocarnitine chloride) for 5 years without experiencing further attacks, and her cognitive and motor development is normal. Further tests on residual enzyme activity, as well as experience with more cases, may shed light on the relationship between gene mutations and phenotypes in MMA.

## Introduction

Methylmalonic acidemia (MMA; MIM# 251000) is an autosomal recessively inherited disorder in which a defect in methylmalonyl CoA mutase (MCM, EC5.4.99.2) results in the build-up of methylmalonic acid and other organic acids in the body. MMA is categorized as either apoenzyme deficiency (*mut*) type or coenzyme B12 metabolic disorder (*Cbl*) type. The *mut* type is subdivided into *mut* ^0^, which results from complete deficiency, and *mut* ^−^, which results from partial deficiency. The *mut* ^0^ type, which is characterized by apoenzyme activity of not more than 0.1 %, often presents with metabolic acidosis within 1 week after birth, frequently resulting in death in early childhood. The *mut* ^−^ type presents with repeated attacks of ketoacidosis triggered by infection after the age of 1 year. MCM is encoded by a single gene, *mut*, which is located on 6p21. *Mut* consists of 13 exons [1–3]. We herein report a case of a girl with MMA in which two missense mutations were found: a novel I739T mutation located in the most C-terminal exon 13 ever reported and the L494X mutation located in exon 8, which is relatively common in Japanese patients.

## Patient Report

A healthy 1.5-year-old girl went to sleep one night after vomiting. In the morning, the parents found the girl unconscious in bed and took her to the emergency room. The girl was 76.3-cm tall and weighed 8.4 kg with a body temperature of 35.2 °C and blood pressure of 77/47 mmHg. She did not open her eyes in response to speech, and the results of simple blood glucose test were below the detectable limit. She regained consciousness following administration of 20 % blood glucose, but experienced tonic convulsions and rolling eyes for several minutes. The convulsions were controlled with phenobarbital. Intravenous fluids were started. The patient was lucid after 3 h, and her motor functions improved after 6 h to the point where she could walk on her own. Blood gas analysis at the time the patient was hospitalized showed pH 7.240, pCO_2_ 41.5 mmHg, base excess −9.2, and HCO_3_ −17.2 mmol, indicating metabolic acidosis. Other abnormal results were lactic acid, 35 mg/dL; NH_3_, 118 μg/dL; AST, 143 U/L; and ALT, 46 U/L. Urine specific gravity was 1.04 with a urine ketone level of 3+.

Organic acid test was performed on suspicion of congenital metabolic anomaly. The results showed a pronounced increase in methylmalonate excretion with increased methylcitrate and 3-OH-propionate excretion, leading to a diagnosis of MMA. Because the approximate urine methylmalonic acid level at onset was 658.4 μM/mmol creatinine, high-dose oral administration of Vitamin B12 (10 mg of cobamamide) was started, resulting in a decrease to 213.0 μM/mmol creatinine on day 7 of treatment.

HPLC quantification of succinyl-CoA production upon reaction of the patient’s lymphocyte lysates with methylmalonyl-CoA and adenosylcobalamin revealed that the patient’s methylmalonyl-CoA activity level was 1.4 pmol succinyl-CoA/min/10^6^ cells, which is approximately 5 % of the level seen in healthy controls (35.9 ± 16.4 pmol succinyl-CoA/min/10^6^ cells).

The patient has now been on high-dose oral administration of Vitamin B12 and carnitine therapy (900 mg of levocarnitine chloride) for 5 years. She has not experienced further attacks; her cognitive and motor development is normal.

## *mut* Genetic Analysis

**Fig. 1:**
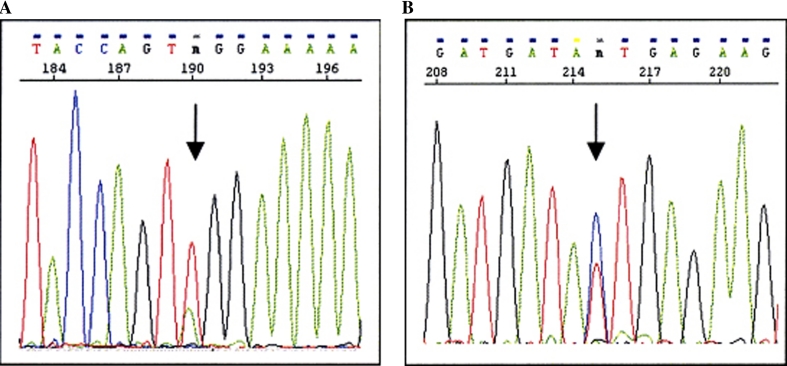
**a** Mutation c.1481T > A (p.L494X) in exon 8 of *MUT* gene. **b** Novel c.2216T > C (p.I739T) mutation in exon 13 of *MUT* gene

Analysis of the *mut* gene was performed for definitive analysis of the type of MMA in this patient. A T-to-A substitution at nucleotide position 1481 in exon 8 of the *mut* gene was found in a heterozygous pattern (Fig. 1a).

A T-to-C substitution at nucleotide position 2216 in exon 13 was also found in a heterozygous pattern. As a result of this base substitution, the amino acid isoleucine at position 739 was changed to threonine, resulting in c.2216T > C (p.I739T). The child was therefore definitively diagnosed with methylmalonic acidemia (*mut*) (Fig. 1b).

## Discussion

In this case of MMA, we found the L494X mutation in exon 8, which is common in Japanese patients, and an I739T mutation in exon 13, which has never previously been reported. The patient in this case presented with convulsions associated with hypoglycemia. The clinical course after hospitalization was relatively mild for MMA. Blood and urinalysis data suggested that this case of MMA was an organic acid metabolic disorder, and the patient was treated with high doses of Vitamin B12 after more detailed analysis. Before the start of Vitamin B12 treatment, the approximate urine methylmalonic acid levels were 658.4 μM/mmol creatinine, as compared with 213.0 μM/mmol creatinine after treatment. This case was therefore originally thought to involve Vitamin B12-reactive MMA. However, HPLC quantification of succinyl-CoA production upon reaction of the patient’s lymphocyte lysates with methylmalonyl-CoA and adenosylcobalamin revealed that the child’s enzyme activity was approximately 5 % of normal, which was not indicative of the B12 reactive type. We therefore performed *mut* genetic analysis. Ultimately, a novel I739T mutation in exon 13 and the L494X mutation in exon 8 were found, resulting in a definitive diagnosis of the *mut* ^−^ type MMA.

Of the 119 mutations of the *mut* gene reported previously, the novel I739T mutation in exon 13 found in this case is the closest to the C terminal [1–5]. The (β, α)5cobalamin (vitamin B12) binding domain occurs in C-terminal exons 10, 11, 12, and 13 of the *mut* gene. An extremely interesting point in this case is that the reaction shown by urine methylmalonic acid levels following clinical administration of Vitamin B12 was consistent with the location of the genetic mutation. This warrants further analysis, including in vitro tests on the expression of residual enzyme activity after the addition of Vitamin B12. Further experience with more cases may shed light on the relationship between gene mutations and phenotypes in MMA.
